# Factors Associated with Adherence to Cardiac Rehabilitation: A Retrospective Cohort Study

**DOI:** 10.3390/jcm15176534

**Published:** 2026-08-24

**Authors:** Jessica Campo-Álvarez, Mónica Rincón-Roncancio, Eduardo Tuta-Quintero, Yuli Fuentes, Claudia Mondragón-Rinta, Liliana García-Gutiérrez

**Affiliations:** 1 Department of Physical Medicine and Rehabilitation, Universidad de La Sabana, Chía 250001, Colombia; campojessica71@gmail.com; 2 Department of Physical Medicine and Rehabilitation, Fundación Cardio Infantil, Instituto de Cardiología, Bogotá 111711, Colombia; mrincon@lacardio.org; 3 Department of Epidemiology, School of Medicine, Universidad de La Sabana, Chía 250001, Colombia; eduardotuqu@unisabana.edu.co (E.T.-Q.); yuli.fuentes@unisabana.edu.co (Y.F.); 4 Department of Physical Medicine and Rehabilitation, Clínica Universidad de La Sabana, Chía 250001, Colombia; claudiamr@clinicaunisabana.edu.co

**Keywords:** cardiac rehabilitation, cardiovascular outcomes, observational study

## Abstract

**Introduction:** Cardiac rehabilitation (CR) is an evidence-based secondary prevention strategy that improves functional capacity, quality of life, and cardiovascular outcomes. However, adherence to CR programs remains suboptimal, limiting their effectiveness. **Methods:** A retrospective multicenter cohort study was conducted among patients enrolled in outpatient CR programs at two specialized cardiovascular centers. Adherence was defined as attendance at ≥70% of the prescribed sessions (≥25 of 36 sessions). Bivariate analyses were performed to compare adherent and non-adherent participants. Variables with *p* < 0.20 were included in a multivariable logistic regression model to identify factors independently associated with adherence. **Results:** A total of 210 participants were included, of whom 77.6% (163/210) were classified as adherent. The median age was 68 years (IQR 60.3–75.0), and 64.3% were men. Most participants (70.5%) completed more than 30 rehabilitation sessions. Adherence rates were highest among patients undergoing surgical myocardial revascularization (80.6%) and valve surgery (76.0%). In multivariable analysis, program duration was the only factor significantly associated with adherence (OR = 4.59; 95% CI: 2.46–8.59; *p* < 0.001). History of dyslipidemia showed a non-significant trend toward higher adherence (OR = 3.29; 95% CI: 0.91–11.90; *p* = 0.069). Educational level, left ventricular ejection fraction, and health insurance affiliation were not independently associated with adherence. **Conclusions:** Adherence to CR was high in this cohort. Longer participation in the program was the only independent predictor of adherence, highlighting the importance of strategies that promote sustained engagement throughout the rehabilitation process.

## 1. Introduction

Cardiovascular diseases are the leading cause of morbidity and mortality worldwide and represent a substantial burden on healthcare systems due to their high prevalence, recurrence, and functional consequences [1,2,3]. Among secondary prevention strategies, cardiac rehabilitation (CR) is recognized as a comprehensive intervention for patients with established cardiovascular disease or those at high cardiovascular risk, aimed at optimizing physical, psychological, and social well-being, facilitating functional reintegration, and reducing the risk of recurrent cardiovascular events [3,4]. CR is a multidimensional program based on clinical assessment, individualized exercise prescription, education on healthy lifestyle behaviors, and psychosocial support, delivered by an interdisciplinary team [5,6,7]. Its implementation has been shown to improve functional capacity, reduce hospitalizations, and decrease mortality, making it a strongly evidence-based recommendation in international cardiovascular management guidelines [4,5,6,7].

Despite its proven benefits, participation in and completion of CR programs remain limited [7,8]. A considerable proportion of patients either do not enroll in these programs or discontinue participation before completion; a situation associated with multiple barriers related to sociodemographic, clinical, economic, and geographic factors [8,9]. Furthermore, there is no universally accepted definition of adherence in the context of cardiac rehabilitation. This concept encompasses not only regular attendance at rehabilitation sessions but also the sustained adoption of healthy lifestyle behaviors, adherence to pharmacological treatment, and active patient engagement in the therapeutic process [9,10,11]. Adherence can therefore be understood as a dynamic process that includes program initiation, continued participation throughout the intervention, and the long-term maintenance of behavioral changes aimed at reducing cardiovascular risk [10,11]. Identifying the factors associated with adherence is essential for developing strategies that optimize participation and improve clinical outcomes in CR programs.

## 2. Methods

### 2.1. Study Design

A retrospective cohort study was conducted. Data were collected through a review of the medical records of patients enrolled in the CR program at Fundación Cardio Infantil—Instituto de Cardiología and Clínica Universidad de La Sabana between January 2022 and January 2024. This study was designed and reported in accordance with the Strengthening the Reporting of Observational Studies in Epidemiology (STROBE) Statement to ensure transparent and comprehensive reporting of observational research [12].

### 2.2. Eligibility Criteria

Patients were eligible for inclusion if they:

- Initiated an outpatient, face-to-face CR program between January 2022 and January 2024.
- Had a physician’s referral to participate in the CR program.
- Completed the initial program assessment.
- Completed the rehabilitation program within the predefined study period.

Patients were excluded if they:

- Lacked access to institutional medical records.
- Had incomplete clinical information required for the study analysis.
- Discontinued the rehabilitation program for reasons unrelated to adherence (e.g., medical contraindications or other circumstances preventing completion of follow-up).
- Were transferred to another rehabilitation modality during the follow-up period.

### 2.3. Clinical Variables

The following variables were collected: age, sex, marital status, occupation, educational level, place of residence, health insurance status, diagnosis, treatment received for myocardial infarction, history of tobacco use, history of hypertension, dyslipidemia, diabetes mellitus, kidney disease, left ventricular ejection fraction, body mass index (BMI), number of CR sessions attended, and time required to complete the program.

The time variable, expressed in months, was calculated from the date of the first rehabilitation session to the date of the last session recorded in the medical record. Additionally, an adherence variable was created based on the results of a survey conducted among CR experts. Patients were classified as adherent if they completed at least 70% of the prescribed sessions. Given a total of 36 scheduled sessions, adherence was defined as attendance at 25 or more sessions.

### 2.4. Sample Size

The minimum sample size was estimated according to the recommendations for multivariable logistic regression models, assuming at least 10 outcome events per candidate predictor variable to ensure stable parameter estimation. Considering the most comprehensive multivariable model with eight independent covariates, a minimum of 80 outcome events (adherent patients) was required [13,14]. Assuming an adherence prevalence of approximately 50%, at least 160 participants would therefore be necessary. The final study included 210 participants, of whom 163 (77.6%) met the adherence criterion, providing substantially more than the minimum number of outcome events required for the planned multivariable analysis. Based on the final sample size and the observed number of outcome events, the study achieved an estimated statistical power greater than 80% (β < 0.20), supporting the adequacy of the sample for the exploratory multivariable analyses [13,14].

### 2.5. Statistical Analysis

Data extraction was performed using a structured form developed in the REDCap platform [15], and data collection was conducted by the principal investigator. Information was temporarily stored in a digital database on a password-protected computer with restricted access, ensuring data anonymization and participant confidentiality. Upon completion of data collection, the database was exported for analysis using Stata version 14 (StataCorp, College Station, TX, USA).

A descriptive analysis of all study variables was initially performed. Continuous variables were summarized using measures of central tendency and dispersion according to their distribution. Normally distributed variables were reported as mean and standard deviation (SD), whereas non-normally distributed variables were described using median and interquartile range (IQR). Normality was assessed using the Shapiro–Wilk test. Categorical variables were summarized as absolute frequencies and percentages [13,14].

Subsequently, a bivariate analysis was conducted using adherence to the CR program (adherent vs. non-adherent) as the dependent variable. Associations between adherence and the sociodemographic and clinical variables of interest were evaluated. Continuous variables were compared between groups using the independent-samples Student’s t-test when the normality assumption was met; otherwise, the nonparametric Mann–Whitney U test was applied. Comparisons of categorical variables were performed using the chi-square test or Fisher’s exact test, as appropriate [13,14].

An exploratory multivariable analysis was performed using a binary logistic regression model, with adherence status as the dependent variable. Independent variables included sociodemographic and clinical characteristics that showed a *p*-value < 0.20 in the bivariate analysis, to control for potential confounding factors. Final model selection was based on parsimony and statistical fit, assessed using the Akaike Information Criterion (AIC). Model calibration was evaluated using the Hosmer–Lemeshow goodness-of-fit test. Results were reported as adjusted odds ratios (ORs) with corresponding 95% confidence intervals (95% CIs), obtained by exponentiating the model coefficients. Statistical significance was established at a two-sided α level of 0.05, and 95% confidence intervals were calculated for all analyses [13,14].

## 3. Results

A total of 210 participants were included in the overall cohort, of whom 77.6% (163/210) were classified as adherent. The median age of the cohort was 68.0 years (IQR: 60.3–75.0), with no significant differences between adherent participants (68.0 [60.0–75.0]) and non-adherent participants (70.0 [61.5–75.0]; *p* = 0.728). Men accounted for 65.6% (107/163) of the adherent group and 59.6% (28/47) of the non-adherent group (*p* = 0.554). Median body weight was 68.0 kg (IQR: 61.0–77.0), median height was 165 cm (IQR: 157–170), and median BMI was 25.2 kg/m^2^ (IQR: 23.0–27.9), with no significant differences between groups (all *p* > 0.05).

Regarding educational attainment, 35.9% (61/170) of participants had completed university education, while 24.1% (41/170) had completed secondary education and 24.1% (41/170) had completed primary education. No significant differences were observed between adherent and non-adherent participants (*p* = 0.195). The median number of CR sessions attended was 36.0 (IQR: 28.0–36.0) in the overall cohort, 36.0 (36.0–36.0) among adherent participants, and 13.0 (7.0–17.5) among non-adherent participants (*p* < 0.001). Similarly, the median duration of participation in the program was 5.0 months (IQR: 4.4–6.2), corresponding to 5.5 months (4.7–6.7) among adherent participants and 2.0 months (0.7–3.6) among non-adherent participants (*p* < 0.001). Comorbidities are described in Table 1.

No significant differences were observed between adherent and non-adherent participants regarding marital status, employment status, or urban residence (all *p* > 0.05). Most participants were married (61.5% [128/208]), employed or economically active (56.4% [97/172]), and resided in urban areas (97.1% [200/206]) (Supplementary Table S1). In the subgroup analysis according to health insurance affiliation, enrollment in the contributory health insurance regime was more frequent among adherent participants (84.0% [136/162]) than among non-adherent participants (59.6% [28/47]) (*p* = 0.001) (Supplementary Table S2).

In the overall cohort (n = 210), most participants completed more than 30 rehabilitation sessions (70.5% [148/210]), whereas 10.0% (21/210) attended between 21 and 30 sessions, 11.4% (24/210) attended between 11 and 20 sessions, and 8.1% (17/210) attended between 0 and 10 sessions (Table 2).

Among patients who underwent surgical myocardial revascularization (n = 36), 80.6% (29/36) completed more than 30 sessions, 13.9% (5/36) attended between 11 and 20 sessions, and 5.6% (2/36) attended between 21 and 30 sessions; no patients attended fewer than 10 sessions. Among those who underwent percutaneous coronary intervention/angioplasty (n = 70), 64.3% (45/70) attended more than 30 sessions, 14.3% (10/70) attended between 0 and 10 sessions, 11.4% (8/70) attended between 11 and 20 sessions, and 10.0% (7/70) attended between 21 and 30 sessions. Among patients with heart failure (n = 26), 69.2% (18/26) completed more than 30 sessions, 15.4% (4/26) attended between 21 and 30 sessions, 7.7% (2/26) attended between 11 and 20 sessions, and 7.7% (2/26) attended between 0 and 10 sessions. Finally, among patients with arrhythmias (n = 30), 66.7% (20/30) completed more than 30 sessions, 16.7% (5/30) attended between 11 and 20 sessions, 10.0% (3/30) attended between 21 and 30 sessions, and 6.7% (2/30) attended between 0 and 10 sessions.

Time spent in the program, measured in months, was the only variable significantly associated with adherence, showing a 4.59-fold increase in the odds of adherence for each increment in exposure time (OR = 4.59; 95% CI: 2.46–8.59; *p* < 0.001) (Table 3). A history of dyslipidemia showed a trend toward association, with an odds ratio of 3.29 (95% CI: 0.91–11.90; *p* = 0.069), although statistical significance was not reached. Educational level was associated with an OR of 0.69 (95% CI: 0.39–1.21; *p* = 0.197), whereas left ventricular ejection fraction showed an OR of 1.04 (95% CI: 0.98–1.10; *p* = 0.241), and health insurance affiliation an OR of 0.59 (95% CI: 0.15–2.33; *p* = 0.451), with none demonstrating statistically significant associations.

## 4. Discussion

This study evaluated adherence to a CR program and the factors associated with adherence in a cohort of patients with cardiovascular disease. Adherence was defined as completion of at least 70% of the prescribed sessions, according to a previously established expert consensus criterion, and was analyzed as a dichotomous variable (adherent vs. non-adherent). The observed adherence rate was 77.6%, which is higher than that reported by Zhang et al. [16], who found an adherence rate of 57.6% in a U.S. population, and higher than that described by Betancourt et al. [17] in a Colombian population, where adherence reached 66.7%. These differences may be explained by variations in the operational definition of adherence, organizational characteristics of rehabilitation programs, and differences in healthcare system contexts across studies [16,17,18].

No statistically significant differences were observed between adherent and non-adherent patients regarding sociodemographic or clinical characteristics, including age, sex, body weight, height, body mass index, educational level, left ventricular ejection fraction, or comorbidities. Median age was similar between groups, and the proportion of men was comparable. Likewise, educational attainment was not associated with adherence, a finding that is consistent with previous studies suggesting that retention in CR may depend more on structural and organizational aspects of the program than on individual educational characteristics [17,18,19].

Time spent in the program, measured in months, was the only variable significantly associated with adherence [20,21]. For each increase in exposure time, the odds of adherence increased by 4.59-fold. This finding is consistent with the observed distribution of completed sessions, as adherent participants reached a median of 36 sessions (IQR: 36–36), whereas non-adherent participants completed a median of 13 sessions (IQR: 7–17.5). Furthermore, 70.5% (148/210) of the overall cohort completed more than 30 sessions, reflecting a high level of program retention.

Across clinical subgroups, the highest proportion of participants completing more than 30 sessions was observed among those with a history of surgical myocardial revascularization (80.6%), followed by patients who underwent valvular procedures (76.0%), those with heart failure (69.2%), arrhythmias (66.7%), and percutaneous coronary revascularization (64.3%). These findings suggest that perceived disease severity or the type of cardiovascular intervention may indirectly influence treatment continuation, although these variables were not included as independent predictors in the multivariable model [20,21].

A history of dyslipidemia showed a trend toward an association with adherence, although statistical significance was not reached. This finding partially agrees with the results reported by Smith et al. [22], who found that dyslipidemia was associated with greater participation in CR programs, possibly because these patients are more frequently exposed to secondary prevention strategies and long-term clinical follow-up. In contrast to previous reports, no significant associations were observed in this cohort between adherence and sex, age, clinical history, or health insurance status. French reported an association between employment status and participation in rehabilitation programs, whereas Dunlay et al. [23] described lower participation rates among patients with diabetes mellitus. Similarly, Parashar reported lower adherence among active smokers, a finding that was not replicated in the present study.

Although no sex-related differences in adherence were observed, men remained overrepresented in the program, a phenomenon widely documented in the literature [24,25,26]. Marra et al. [27] reported lower participation rates among women, attributed to barriers such as family responsibilities, logistical constraints, transportation dependence, and limited scheduling flexibility. Differences between the present findings and those of previous studies may be partially explained by the heterogeneity in adherence definitions. While adherence in the current study was defined as attendance at ≥70% of prescribed sessions, other investigations have considered only program enrollment or have used lower attendance thresholds, such as 50% of scheduled sessions, limiting direct comparability [27,28].

Adherence in CR is a dynamic process that develops throughout follow-up, and longer participation may both facilitate and reflect sustained engagement with the rehabilitation program [29,30]. Because adherence was defined as a binary outcome based on completion of the prescribed sessions, the present study was designed to identify factors associated with adherence status rather than to evaluate the timing or rate of program dropout. Consequently, the observed association does not imply a causal relationship, and future longitudinal studies using time-to-event approaches may provide additional insight into the temporal dynamics of adherence and attrition in CR programs [30,31,32].

### Limitations

Several limitations should be acknowledged. First, the retrospective design restricts control over potential confounding variables and may introduce selection bias. Second, some population subgroups had limited representation, particularly women and participants affiliated with certain health insurance schemes. Although the sample size was determined according to Freeman’s recommendation for logistic regression modeling and the study included enough adherence events (n = 163) relative to the variables evaluated, no formal a priori statistical power calculation was performed. Therefore, the possibility of limited power to detect small associations for some variables cannot be completely excluded.

The exclusion of patients with incomplete clinical information or unavailable institutional medical records may have introduced selection bias. Individuals with fragmented documentation or limited follow-up could differ systematically from those included in the analysis, potentially reflecting lower engagement with healthcare services and rehabilitation programs. Consequently, the observed adherence rates may not be fully representative of all patients referred to cardiac rehabilitation, and the generalizability of the findings should be interpreted with caution.

Another limitation is that nearly all participants resided in urban areas, with very limited representation of rural populations. Consequently, the findings may not be fully generalizable to patients living in rural or remote settings, where barriers to CR participation and adherence may differ substantially. Finally, although adherence was defined according to expert consensus, this definition primarily reflects administrative compliance with treatment and does not capture patient-centered dimensions such as motivation, perceived barriers, or satisfaction with the rehabilitation program. These findings highlight the need to strengthen institutional strategies aimed at maintaining patient engagement from the earliest stages of CR to maximize the clinical and functional benefits associated with sustained participation.

## 5. Conclusions

Adherence to the CR program was generally high in this cohort. A longer duration of participation in the program was associated with greater adherence, suggesting that sustained engagement may contribute to program completion. No statistically significant associations were observed between adherence and the sociodemographic, clinical, or functional variables evaluated. These findings highlight the potential importance of maintaining participation throughout the rehabilitation process, although further research is needed to better understand other factors that may influence adherence.

## Figures and Tables

**Table 1:** Baseline characteristics of patients according to adherence to the CR program.

	Total Population n = 210	Adherent (n = 163)	No Adherent (n = 47)	*p* Value
Age, me [IQR]	68.0 [60.3–75.0]	68.0 [60.0–75.0]	70.0 [61.5–75.0]	0.728
Male, n (%)	135 (64.3)	107 (65.6)	28 (59.6)	0.554
Weight, me [IQR]	68.0 [61.0–77.0]	67.0 [61.0–77.0]	69.0 [61.0–74.50]	0.765
Height, me [IQR]	165 [157–170]	165 [156–170]	165 [159–169]	0.924
BMI, me [IQR]	25.2 [23.0–27.9]	25.1 [23.1–28.2]	25.3 [22.6–27.7]	0.866
Educational level, n (%)				0.195
No schooling	9 (5.3)	9 (6.3)	0 (0.0)	
Primary school	41 (24.1)	38 (26.8)	3 (10.7)	
Secondary school	41 (24.1)	32 (22.5)	9 (32.1)	
Technical/Technological school	18 (10.6)	14 (9.9)	4 (14.3)	
University studies	61 (35.9)	49 (34.5)	12 (42.9)	
Number of sessions, me [IQR]	36.0 [28.0–36.0]	36.0 [36.0–36.0]	13.0 [7.0–17.5]	<0.001
Time to sessions (months), me [IQR]	5.0 [4.4–6.2]	5.5 [4.7–6.7]	2.0 [0.7–3.6]	<0.001
Comorbidities, n (%)				0.607
AMI	129 (61.4)	98 (60.1)	31 (66.0)	
Valvular heart disease	25 (11.9)	21 (12.9)	4 (8.5)	
Heart failure	26 (12.4)	22 (13.5)	4 (8.5)	
Arrhythmias	30 (14.3)	22 (13.5)	8 (17.0)	
MI treatment, n (%)				0.382
Medical	23 (17.8)	18 (18.4)	5 (16.1)	
Surgical	36 (27.9)	30 (30.6)	6 (19.4)	
Percutaneous	70 (54.3)	50 (51.0)	20 (64.5)	
Smoking, n (%)	45 (21.4)	31 (19.0)	14 (29.8)	
Hypertension, n (%)	119 (56.7)	91 (55.8)	28 (59.6)	
Diabetes mellitus, n (%)	46 (21.9)	38 (23.3)	8 (17.0)	
Kidney disease, n (%)	14 (6.7)	11 (6.7)	3 (6.4)	
Dyslipidemia, n (%)	83 (39.5)	70 (42.9)	13 (27.7)	
LVEF, me [IQR]	55 [45–60]	55 [45–60]	57.5 [50–61]	0.107
LVEF Category, n (%)				0.188
<30	9 (4.4)	8 (5.0)	1 (2.2)	
30–50	68 (33.0)	57 (35.6)	11 (23.9)	
>50	129 (62.6)	95 (59.4)	34 (73.9)	

Notes: BMI: Body mass index, IQR: Interquartile range, LVEF: Left ventricular ejection fraction, AMI: Acute myocardial infarction.

**Table 2:** Number of CR sessions by diagnosis.

Number of Sessions, n (%)	Overall Cohort (n = 210)	Surgical Myocardial Revascularization (n = 36)	Percutaneous Revascularization (Angioplasty) (n = 70)	Acute Myocardial Infarction (n = 23)	Valve Surgery (n = 25)	Heart Failure (n = 26)	Arrhythmias (n = 30)
0–10	17 (8.1)	0 (0)	10 (14.3)	3 (13.0)	0 (0)	2 (7.7)	2 (6.7)
11–20	24 (11.4)	5 (13.9)	8 (11.4)	2 (8.7)	2 (8.0)	2 (7.7)	5 (16.7)
21–30	21 (10.0)	2 (5.6)	7 (10.0)	1 (4.3)	4 (16.0)	4 (15.4)	3 (10.0)
>30	148 (70.5)	29 (80.6)	45 (64.3)	17 (73.9)	19 (76.0)	18 (69.2)	20 (66.7)

**Table 3:** Logistic regression model for the dependent variable of adherence.

Variable	OR	95% CI	*p*-Value
Educational level	0.69	0.39–1.21	0.197
History of dyslipidemia	3.29	0.91–11.90	0.069
Left ventricular ejection fraction	1.04	0.98–1.10	0.241
Duration in the program	4.59	2.46–8.59	<0.001
Health insurance affiliation	0.59	0.15–2.33	0.451

## Data Availability

The original contributions presented in this study are included in the article. Further inquiries can be directed to the corresponding author.

## Supplementary Materials

The following supporting information can be downloaded at: https://www.mdpi.com/article/10.3390/jcm15176534/s1, Table S1: Comparison of sociodemographic characteristics between adherent and non-adherent patients; Table S2: Sub-analysis by type of social security affiliation between contributory versus complementary/prepaid/private plan.
