# Supplementary material for: Factors Associated with Adherence to Cardiac Rehabilitation: A Retrospective Cohort Study
Source: J Clin Med. 2026 Aug 24;15(17):6534. doi: 10.3390/jcm15176534 (PMC13566034; doi:10.3390/jcm15176534)
Supplement: Supplementary file 1 [file jcm-15-06534-s001.zip › jcm-4398648-supplementary.pdf]

Supplementrary Table S1. Comparison of sociodemographic characteristics between adherent and non-adherent patients

Characteristics	General Cohort (n=210)	Adherent (n=163)	No Adherent (n=47)
Marital status, n (%)			
Single	50 (24.0)	40 (24.8)	10 (21.3)
Married	128 (61.5)	97 (60.2)	31 (66.0)
Common-law union	19 (9.1)	16 (9.9)	3 (6.4)
Widowed	11 (5.3)	8 (5.0)	3 (6.4)
Occupation, n (%)			
Employed	97 (56.4)	71 (55.0)	26 (60.5)
Unemployed	18 (10.5)	13 (10.1)	5 (11.6)
Unemployed	57 (33.1)	45 (34.9)	12 (27.9)
Urban origin, n (%)	200 (97.1)	156 (96.3)	44 (100.0)
Health insurance, n (%)			
Subsidized	1 (0.5)	1 (0.6)	0 (0.0)
Contributory	164 (78.1)	136 (83.4)	28 (59.6)
Private/Prepaid	45 (21.4)	26 (16.0)	19 (40.4)

Supplementrary Table S2. Sub-analysis by type of social security affiliation between contributory versus complementary/prepaid/private plan.

Characteristics of social security affiliation, n (%)	Adherent (n=162)	No adherent (n=47)	*p-value*
Contributory	136 (84.0)	28 (59.6)	0.001
Supplementary/Prepaid/Private Plan	26 (16.0)	19 (40.4)	
